# Modulation of gentamicin-induced acute kidney injury by *myo*-inositol oxygenase via the ROS/ALOX-12/12-HETE/GPR31 signaling pathway

**DOI:** 10.1172/jci.insight.155487

**Published:** 2022-03-22

**Authors:** Isha Sharma, Yingjun Liao, Xiaoping Zheng, Yashpal S. Kanwar

**Affiliations:** Departments of Pathology and Medicine, Feinberg School of Medicine, Northwestern University, Chicago, Illinois, USA.

**Keywords:** Nephrology, Molecular pathology

## Abstract

In this investigation, a potentially novel signaling pathway in gentamicin-induced acute kidney injury—worsened by overexpression of proximal tubular enzyme, *myo*-inositol oxygenase (MIOX)—was elucidated. WT, MIOX-transgenic (MIOX-Tg), and MIOX-KO mice were used. Gentamicin was administered to induce tubular injury. MIOX-Tg mice had severe tubular lesions associated with increased serum creatinine and proteinuria. Lesions were relatively mild, with no rise in serum creatinine and no albuminuria in MIOX-KO mice. Transfection of HK-2 cells with MIOX-pcDNA led to increased gentamicin-induced reactive oxygen species (ROS). Marked increase of ROS-mediated lipid hydroperoxidation was noted in MIOX-Tg mice, as assessed by 4-HNE staining. This was associated with increased expression of arachidonate 12-lipoxygenase (ALOX-12) and generation of 12-hydroxyeicosatetraenoic acid (12-HETE). In addition, notable monocyte/macrophage influx, upregulation of NF-κB and inflammatory cytokines, and apoptosis was observed in MIOX-Tg mice. Treatment of cells with ALOX-12 siRNA abolished gentamicin-mediated induction of cytokines and 12-HETE generation. HETE-12 treatment promoted this effect, along with upregulation of various signaling kinases and activation of GPCR31. Similarly, treatment of cells or mice with the ALOX-12 inhibitor ML355 attenuated inflammatory response, kinase signaling cascade, and albuminuria. Collectively, these studies highlight a potentially novel mechanism (i.e., the ROS/ALOX-12/12-HETE/GPR31 signaling axis) relevant to gentamicin-induced nephrotoxicity modulated by MIOX.

## Graphical abstract

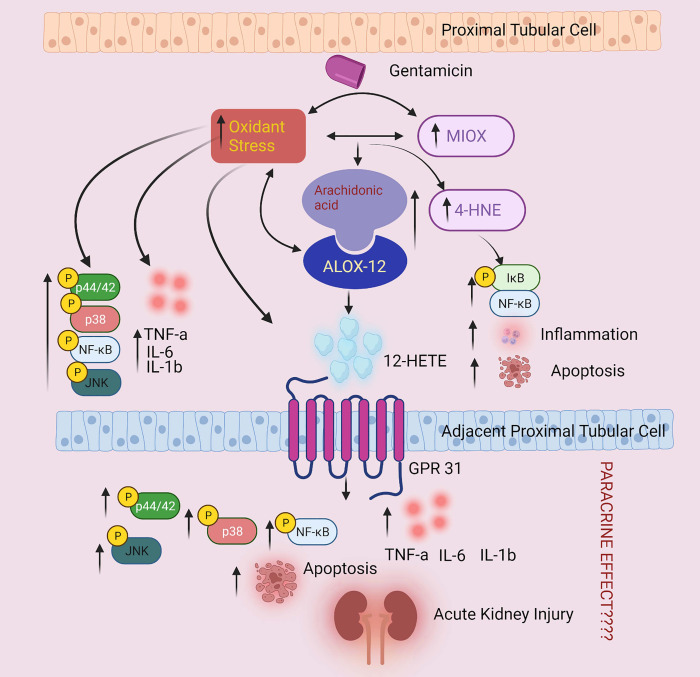

## Introduction

Acute kidney injury (AKI) is commonly referred to as a state in which there is a rapid decline of renal functions that are reflected in the deterioration of glomerular filtration rate (GFR) and a rise in blood levels of nitrogenous products like creatinine and urea nitrogen. The overall incidence of AKI may vary from 5% to 15% in hospitalized patients (1, 2). The inception of AKI may be due to prerenal, renal, or postrenal causes, and in the majority (35%–70%) of patients, the etiology per se is intrinsically confined to the kidney. In the kidney, various compartments that may be afflicted in AKI include tubular, glomerular, interstitial, and vascular compartments. The tubular compartment—especially the proximal tubules, being metabolically active—are highly amenable to ischemic or nephrotoxic injury (1, 3). The latter may be due to the agents exogenously administered, such as aminoglycosides, like gentamicin (GEN) and cis-platinum, or such as those produced endogenously, like hemoglobin and myoglobin. Numerous studies focusing on the tubular compartment have been carried out over several decades, utilizing various animal models to investigate the pathogenesis of human AKI (4). Among various models, ischemia- and cisplatin-induced tubular injury are the 2 major animal model systems that were utilized to delineate a number of diverse pathogenetic mechanisms relative to AKI, and this has been the subject of recent reviews (5–7).

In contrast to the voluminous literature on ischemia or cisplatin-induced tubular injury, the available information relating to the pathogenetic mechanisms of GEN-induced nephrotoxicity in various animal model systems is somewhat limited (8, 9). GEN belongs to the family of aminoglycosides. Apparently, like other aminoglycosides, it is a polycationic molecule that poorly crosses the plasmalemma and remains in the proximal tubular lumina after having undergone glomerular ultrafiltration (9, 10). A small amount of aminoglycosides in the tubular retentate binds to the anionic phospholipids of the proximal tubular brush membrane, especially of the S1 and S2 segments, where it is taken up by adsorptive endocytosis to be routed into the lysosomes to induce phospholipidosis, characterized by multilaminated myelin figures, and ultimately renal cellular toxicity (9, 10). GEN is used often in clinical practice to treat Gram-negative infections; thus, nephrotoxicity is an unavoidable consequence, and the incidence of GEN-induced AKI could vary from 8% to 26% (11, 12). Nephrotoxicity is associated even with a low-dose administration of GEN for 4–5 days. The major brunt of the cellular damage is confined to the renal cortex, where it is internalized by the proximal tubular cells following the interaction of polybasic GEN with megalin, a high molecular mass (M_r_) (~600 kDa) transmembrane protein that serves as a scavenger receptor (13).

In regard to the mechanisms of GEN-induced toxicity, cellular alterations in the form of lysosomal phospholipidosis have been well-described (9, 10). In this process, there is an accumulation of GEN in the lysosomes and inhibition of phospholipases A1 and C, as well as sphingomyelinase with the formation of intracytoplasmic myeloid bodies (9, 10, 14). Another cellular change that has been reported pertains to the inflammatory processes affecting the tubulo-interstitial compartment (15). In terms of relevant biochemical or signaling mechanisms, they include accentuated lipoperoxidation and excessive generation of oxygen radicals, thus rendering renal cortical cells to oxidant stress and, ultimately, apoptosis (14–16). Besides oxidant stress, another mechanism where acceleration of renal injury secondary to other causes includes capillary rarefaction of the tubulo-interstitial compartment due to “repeated episodes of AKI” (17, 18). This process ultimately leads to hypoxia and increased transcription of hypoxia-inducible factor α (HIF-α) (17, 19). In fact, there is an intricate relationship between renal hypoxia and oxidant stress, each mutually boosting the activity of one another, resulting in the aggravation of cellular injury and, conceivably, progression toward chronic kidney disease (CKD) (19, 20). Thus, the consensus of various studies mentioned above is that GEN mainly affects the biology of the tubulo-interstitial compartment, and conceivably, the major cellular target of the oxidant stress seems to be mainly confined to the proximal tubular epithelia.

In view of the above, we ventured to search for a molecule that is expressed in the proximal tubular epithelia and that can modulate the GEN-induced oxidant stress in this particular compartment of the nephron. One type of this molecule is an enzyme known as *myo*-inositol oxygenase (MIOX), which is exclusively expressed in the proximal tubular epithelia, and its expression affects the status of cellular redox in these cells, as described in various model systems—e.g., diabetic nephropathy and cisplatin-induced cellular injury (21–23). With this premise in perspective, we employed various strains of mice with overexpression of MIOX or its gene disruption to comprehensively assess the status of tubular injury, cellular redox, and other unexplored biological signaling mechanisms that may be relevant to the pathobiology of GEN-induced AKI.

## Results

This investigation describes a seminal role of lipoxygenase-12 pathway in GEN induced AKI. Interestingly, it also seems that MIOX overexpression accentuates AKI. We observed that GEN administration increased arachidonate 12-lipoxygenase (ALOX-12) expression, which in turn accentuated 12-hydroxyeicosatetraenoic acid/GPCR31 (12-HETE/GPR31) signaling. Overall, this study unravels a perturbed cellular signaling pathway involving the ALOX-12/12-HETE/GPR31 axis that modulates the progression of GEN-induced AKI.

### MIOX overexpression accentuates GEN induced acute tubular injury in MIOX-transgenic (MIOX-Tg) mice.

**Figure 1:**
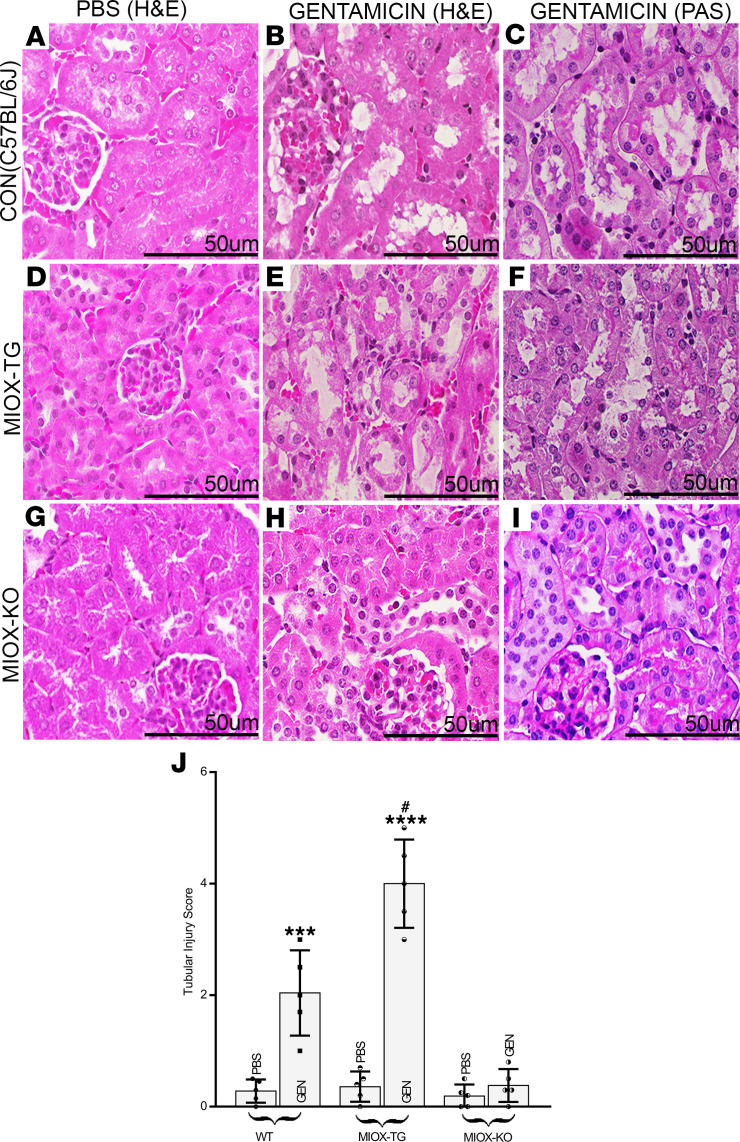
Accentuation of gentamicin induced acute tubular injury following MIOX overexpression. Kidneys of WT control mice revealed moderate cytolysis drop out of nuclei and patchy loss of tubular brush borders following gentamicin (GEN) administration (**A**–**C**). A remarkable deterioration of tubular epithelial morphology with notable cytolysis, loss of brush border and attenuation of tubular basement membranes (TBMs) in MIOX-transgenic (MIOX-Tg) mice was observed (**D**–**F**). The MIOX-KO mice had minimal disruption in tubular morphology following gentamicin administration, as compared with kidneys of WT and MIOX-Tg mice (**G**–**I**). Scale bars: 50 μm. The graph in **J** shows a n increase in tubular injury in MIOX-Tg mice as compared with WT and MIOX-KO mice. (*n* = 5; ***P ≤ 0.001, ****P ≤ 0.00001, compared with control PBS group; ^#^*P* ≤ 0.01, compared with GEN groups; 1-way ANOVA with Dunn’s multiple-comparison test).

GEN sulfate (100 mg/kg) or PBS was administrated i.p. to WT, MIOX-Tg, and MIOX-KO (null) mice. GEN induced a moderate deterioration in tubular epithelial morphology in WT mice in the form of nuclei dropout and cytolysis (Figure 1, A–C). The treated mice had a certain degree of patchy loss of tubular epithelial brush borders, as evident by PAS staining (Figure 1C). However, the morphology of the tubular basement membranes (TBMs) remained clearly distinct. The GEN treatment led to a marked deterioration of cortical tubular morphology with substantial cytolysis of the epithelia in MIOX-Tg mice (Figure 1, D–F). Besides, the treated mice had a notable loss of brush border of the tubular epithelia (Figure 1F), and the outlines of the TBMs became poorly distinct, suggesting a notable cellular disruption. The MIOX-KO mice administered with GEN revealed minimal cellular disruption in tubular morphology (Figure 1, G–I). No significant cellular cytolysis was observed. Interestingly, the morphology of TBMs and brush borders remained preserved in most of the tubules, in contrast to WT and MIOX-Tg mice (Figure 1I). The tubular injury score among various strains of mice following GEN treatment are included in Figure 1J.

**Figure 2:**
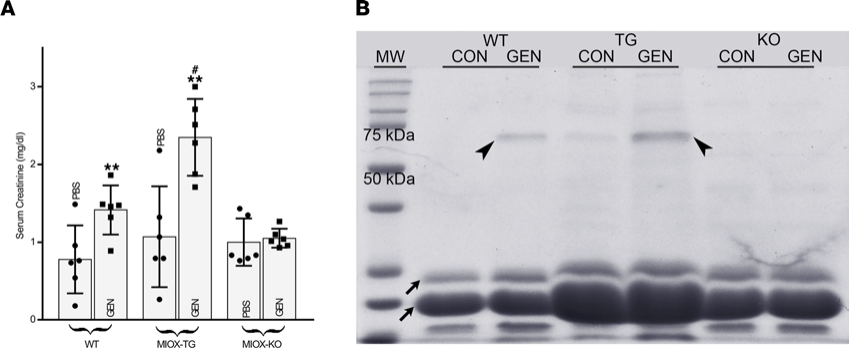
Gentamicin administration worsens serum creatinine levels and exacerbates proteinuria. (**A **and** B**) A notable rise is shown in serum creatinine levels and proteinuric response (arrow and arrowheads, respectively) in MIOX-Tg mice as compared with WT and MIOX-KO mice. (*n* = 3 independent experiments with 2 duplicates each; **P ≤ 0.01, compared with control PBS groups; ^#^*P* ≤ 0.01, compared with GEN groups; 1-way ANOVA with Dunn’s multiple-comparison test).

Parallel changes were seen in the renal physiological parameters in various strains of mice. The serum creatinine levels increased following GEN administration. A moderate increase in its levels was observed in WT mice (Figure 2A). A very mild increase was observed in MIOX-KO mice, whereas a notable increase (~2 folds) in creatinine levels was observed in MIOX-Tg mice following GEN administration. The changes were also reflected in the urinary excretion of albumin, as assessed by SDS-PAGE analyses (Figure 2B, arrowheads). A notable albuminuria was observed following GEN injection in WT mice, while no significant excretion was observed in MIOX-KO. On the other hand, a remarkable degree of albuminuria, much more than WT mice, was observed in MIOX-Tg mice following the GEN treatment. The increased albuminuria in MIOX-Tg was also accompanied with low molecular proteinuria (Figure 2B, arrows), suggesting cellular damage to the tubules in this strain of mice that apparently may be related to the overexpression of MIOX.

### MIOX overexpression accentuates GEN-induced oxidant stress in HK-2 cells.

**Figure 3:**
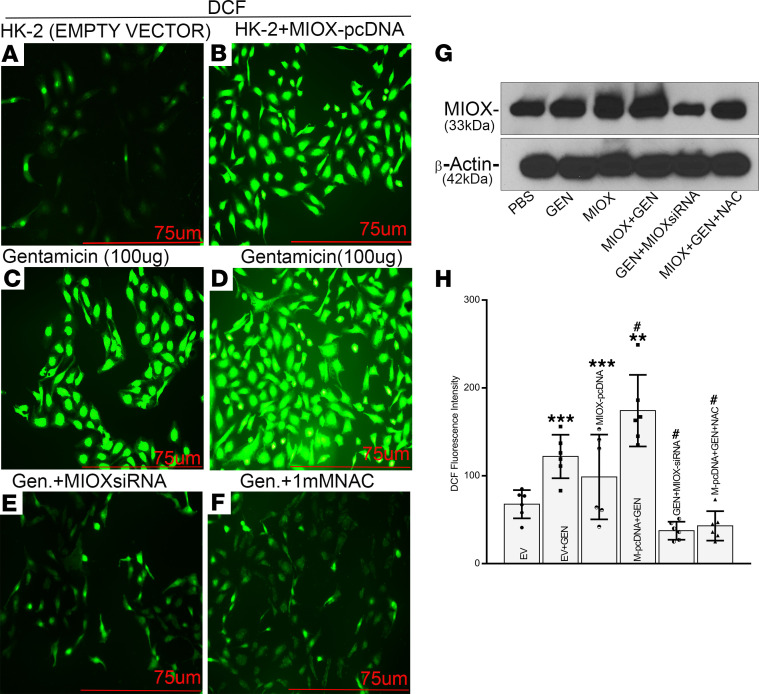
Augmentation of gentamicin-induced oxidant stress by MIOX overexpression in HK-2 cells. Gentamicin-treated HK-2 cells, a proximal tubular cell line, showed increased DCF staining, suggesting perturbation in the redox potential (**A** and** C**). Transfection of MIOX-pcDNA also induced an increase in the generation of reactive oxygen species (ROS) (**A** and** B**). Treatment of MIOX-overexpressing cells with gentamicin led to a marked increase in the intensity of DCF staining (**B–D**). Concomitant treatment of gentamicin with MIOX-siRNA or N-acetyl L-cysteine (NAC) led to a remarkable decrease in the DCF staining (**C–F**). Scale bars: 75 μm.(**G**) Differential increase in MIOX expression. MIOX-overexpressing cell with gentamicin treatment increases MIOX expression, which goes down with siRNA and NAC treatment, reflecting concomitant increase in ROS generation. (**H**) Quantitation of ROS generation with different treatments of HK-2 cells (*n* = 3 independent experiments with 2 duplicates each; ** *P* ≤ 0.01, ***P ≤ 0.001, compared with control EV groups; ^#^*P* ≤ 0.05, compared with GEN groups; 1-way ANOVA with Dunn’s multiple-comparison test).

To delineate if the tubular damage is directly related to the GEN/MIOX-induced oxidant stress, initial in vitro experiments with HK-2 cells, a proximal tubular cell line, were carried out. The GEN-treated HK-2 cells showed perturbation in redox potential, as indicated by the increased 2’-7’dichlorodihydrofluroescein diacetate (DCF) staining (Figure 3, A and C). Interestingly, the overexpression of MIOX—i.e., transfection of MIOX-pcDNA per se—also induced an increase in the generation of reactive oxygen species (ROS), a finding that is similar to our previous observations (Figure 3, A and B) in other model systems (21, 23). The treatment of MIOX-overexpressing cells with GEN led to a further increase in the intensity of DCF staining, suggesting an accentuated generation of ROS (Figure 3, B–D). To confirm if the MIOX overexpression contributes to perturbed redox potential, the cells were concomitantly treated with MIOX-siRNA or N-acetyl L-cysteine (NAC). This led to a remarkable decrease in the DCF staining, suggesting that MIOX itself contributes to the generation of ROS and that it accentuates oxidant stress following GEN treatment (Figure 3, C–F and H). Increase and decrease in ROS generation was dependent on MIOX expression, as reflected by immunoblotting of MIOX in HK-2 cells undergoing various treatments (Figure 3G). MIOX-overexpressing cells treated with GEN showed a notable increase in MIOX expression, which was downregulated by MIOX siRNA and NAC treatment.

### MIOX overexpression augments GEN-induced lipid hydroperoxidation, ALOX-12 expression, and 12-HETE generation.

To understand the mechanism by which GEN induces tubular cell damage, experiments related to lipid hydroperoxidation were initiated since DCF staining suggested that MIOX overexpression exacerbates GEN-induced oxidant stress in HK-2 cells (Figure 3).

**Figure 4:**
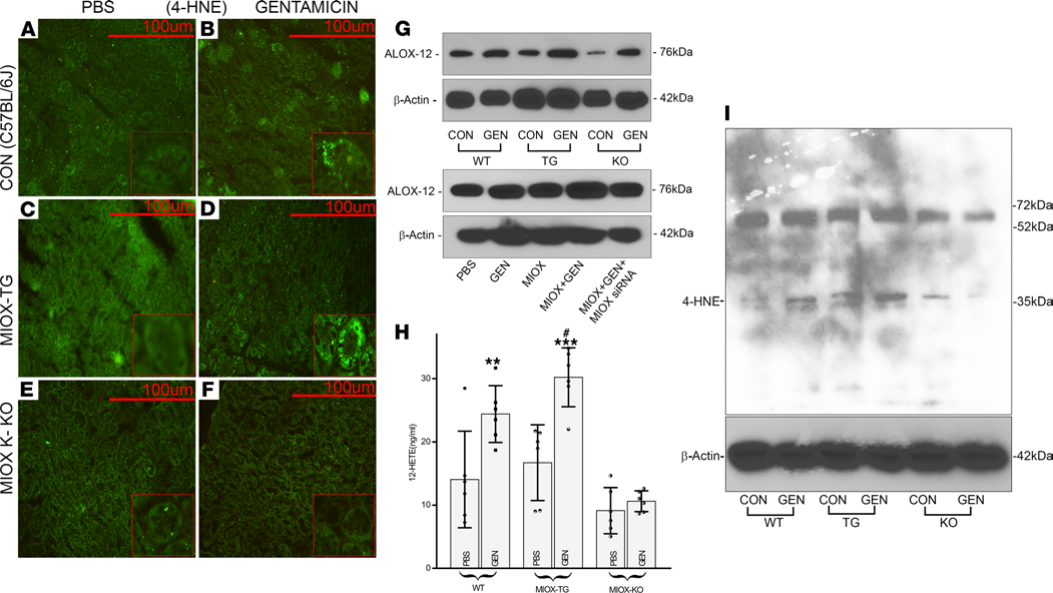
Augmentation of gentamicin-induced lipid hydroperoxidation, ALOX-12 expression, and 12-HETE generation following MIOX overexpression. WT and MIOX-KO mice showed background immunofluorescence (IMF) staining for 4-HNE, a product of lipid peroxidation (**A **and** E**). A mild increase in the intensity of IMF staining and granularity of the tubular cytoplasm was seen following gentamicin treatment (**B**, inset). A substantial increase in the intensity of IMF along with notable granularity of the cytoplasm was observed in the tubules of MIOX-Tg mice administered with gentamicin, suggesting accentuated lipid peroxidation (**C** and **D**, inset). The MIOX-KO mice had minimal IMF staining (**E **and** F**). Scale bars: 100 μm. Immunoblotting revealed a remarkable increase in the expression of ALOX-12, an enzyme that converts AA (poly-unsaturated fatty acid, PUFA) to 12-HETE, in MIOX-Tg mice (upper panel,** G**) and in MIOX overexpressing HK-2 cells (lower panel,** G**) treated with gentamicin. Along with the increase in ALOX-12 protein expression, there was a synchronous increase in the generation of 12-HETE, especially in MIOX-Tg mice receiving gentamicin (**H**) (*n* = 3 independent experiments with 2 duplicates each; **P ≤ 0.01, ***P ≤ 0.001, compared with control PBS groups; ^#^*P* ≤ 0.05, compared with GEN groups; 1-way ANOVA with Dunn’s multiple-comparison test). (**I**) The immunoblot analysis of 4-HNE in various strains of mice. Gentamicin-treated mice had a considerable increase in lipid peroxidation of proteins with a lower molecular weight, with a maximum increase in MIOX-Tg mice administered gentamicin.

Increased ROS generation is likely to lead to oxidative damage of the plasmalemmal lipids, especially those with multiple carbon double bonds—e.g., polyunsaturated fatty acids (PUFAs)—conceivably a signaling molecule that activates GPCRs (24, 25). In view of this, we assessed the status of 4-hydroxynonenal (4-HNE), which is one of the major end products of lipid peroxidation, besides malondialdehde (MDA). The 4-HNE is a known signaling molecule at its “low or medium” concentrations and activates several transcription factors that regulate diverse biological processes (24, 25). The expression of 4-HNE was evaluated by using immunofluorescence (IMF) microscopy. WT and MIOX-KO mice showed background staining for 4-HNE (Figure 4, A and E). WT mice following GEN administration showed moderate damage to lipids, as indicated by a mild increase in the intensity of IMF staining and granularity of the tubular cytoplasm (Figure 4B, inset). A remarkable increase in 4-HNE, seen via staining, along with notable granularity of the cytoplasm, was observed in renal tubular compartments of MIOX-Tg mice administered GEN, suggesting accentuated lipid hydroperoxidation (Figure 4, C and D, inset). However, the mice with MIOX genetic deletion had minimal IMF staining, suggesting no significant lipid peroxidation following GEN administration (Figure 4F). The changes in 4-HNE IMF in kidneys of various strains of mice were also reflected in Western blotting studies (Figure 4I). Notably, the kidneys of MIOX-Tg mice had a relatively increased intensity of 4-HNE bands as compared with others. To affirm the 4-HNE staining was proximal tubule specific, they were stained with MIOX, which is exclusively present in proximal tubules (Supplemental 1, A–C).

One of the PUFA is arachidonic acid (AA), and previous investigations have suggested that AA metabolism is adversely affected in hypoxia-mediated hepatic injury (26). Using the integrative omics approach, Zhang et al. noted that, among the differentially expressed genes responsible for AA metabolism, the *Alox12,* encoding lipoxygenase, was the major upregulated gene that primarily converted AA to 12-HETE (26). We attempted to address the question of whether MIOX overexpression augments the GEN-induced ALOX-12 expression and generation of its primary metabolite (i.e., 12-HETE). To explore the relevance of this hypothesis, we assessed ALOX-12 protein expression in various strains of mice. In general, GEN increased the expression of ALOX-12 in mice (Figure 4G, upper panels). Although the increase was notable in WT and KO, a remarkable upregulation of ALOX-12 was seen in MIOX-Tg mice administered with GEN, suggesting that MIOX does contribute to the modulation of lipoxygenase. Similar results were observed in vitro experiments with HK-2 cells. A notable increase in the ALOX-12 expression was observed in MIOX-overexpressing cells treated with GEN, and this upregulation was normalized following concomitant transfection of MIOX-siRNA (Figure 4G, lower panels), thus confirming the modulatory role of MIOX in the lipoxygenase pathway.

One of the derivatives of AA is 12-HETE, which is produced by the action of ALOX-12. In this context, we initiated studies to test the activity of lipoxygenase by measuring the generation of 12-HETE. Interestingly, the generation of 12-HETE was comparable with the ALOX-12 protein expression modulated by GEN and MIOX (Figure 4, G and H). Overall, the GEN administration increased the generation of 12-HETE in WT and KO mice. The most remarkable increase in the 12-HETE generation was observed in MIOX-Tg mice receiving GEN, thus establishing the fact that MIOX-initiated heightened signaling has indeed affected the ALOX-12 pathway. Ultimately, these sequence of events possibly could affect the expression of inflammatory cytokines, since 4-HNE modulates several transcription factors, including, NF-κB, which is a dimeric transcription factor that modulates a number of pathobiological processes, such as immune responses, inflammation, cell proliferation, and apoptosis (24, 27, 28).

### MIOX overexpression heightens the GEN-induced inflammatory responses and expression of related molecules.

**Figure 5:**
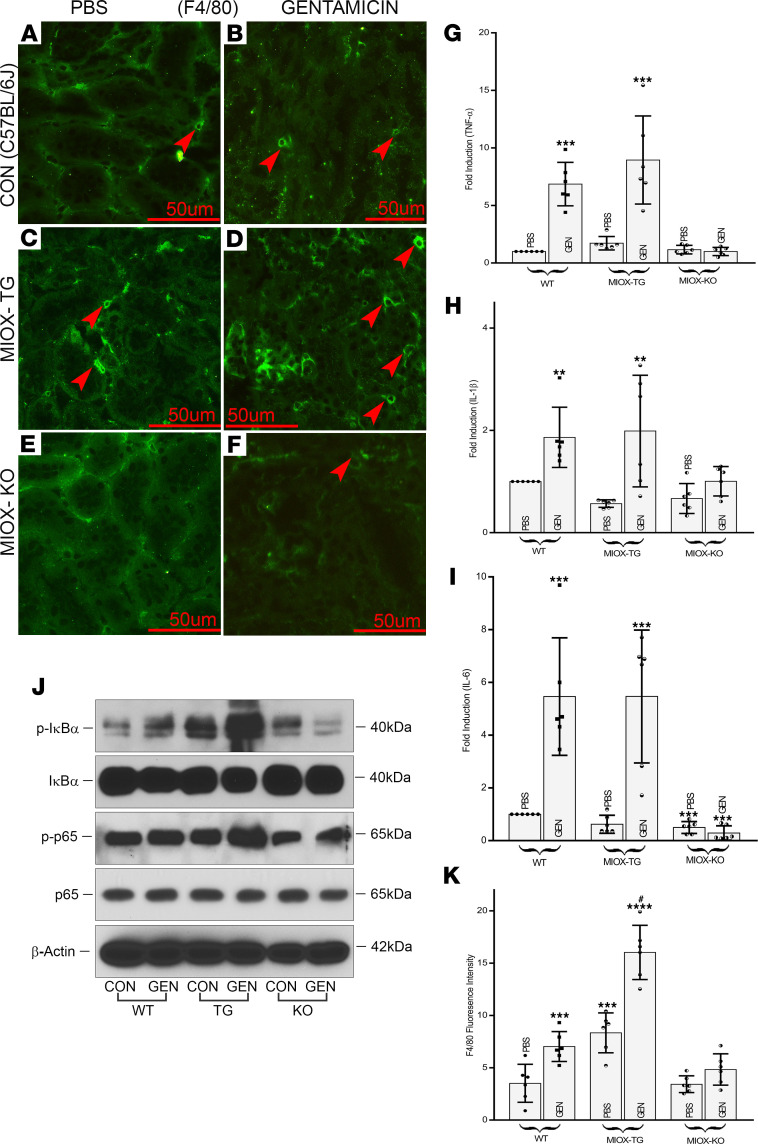
Enhancement of gentamicin-induced inflammatory responses and expression of related molecules following MIOX overexpression. A mild increase in the macrophage population, stained with anti-F4/80 antibody, was observed in kidneys of WT mice treated with gentamicin (**A **versus** B**, arrowheads). A very mild increase in their population was also seen in MIOX-KO mice (**E **versus** F**, arrowheads). Whereas, a significant increase was noted in MIOX-Tg mice (**C **versus** D**, arrowheads). Scale bars: 50 μm. The macrophage population increase was associated with a synchronous enhanced mRNA expression of some of the inflammatory cytokines, including TNF-α, IL-1β, and IL-6, following gentamicin treatment (**G**–**I**) (*n* = 3 independent experiments with 2 duplicates each; **P ≤ 0.01, ***P ≤ 0.001 compared with control PBS groups; 1-way ANOVA with Dunn’s multiple-comparison test). In general, among various strains, a notable augmented expression of cytokines was observed in kidneys of MIOX-Tg mice. Likewise, following gentamicin treatment, an increased expression and phosphorylation of regulatory molecules associated with the pleiotropic transcription factor NF-κB was observed, and they included p-IκBα and p-p65, as assessed by immunoblotting procedures (**J**). This increase was especially noticeable in kidneys of MIOX-Tg mice. (**K**) Quantitation of F4/80 fluorescence in various strains of mice (*n* = 3 independent experiments with 2 duplicates each; *** *P* ≤ 0.001, ****P ≤ 0.0001, compared with control PBS groups; ^#^*P* ≤ 0.001, compared with GEN groups; 1-way ANOVA with Dunn’s multiple-comparison test).

As noted above, there is an amplified upsurge in the GEN-induced expression of ALOX-12 and generation of 4-HNE and12-HETE in the MIOX-Tg mice; it is likely that there will be an augmented inflammatory cellular response and altered expression of relevant cytokines and transcription factors. In view of this, we initially assessed the influx of macrophages within the tubulo-interstitium, since they represent one of the indices of inflammation. Their renal localization was delineated by IMF staining with anti-F4/80 antibody. A mild increase in their population was observed in WT mice treated with GEN (Figure 5, A, B, and K). Likewise, a very mild increase was also observed in MIOX-KO mice (Figure 5, E F, and K). However, a remarkable increase was observed in MIOX-Tg mice undergone GEN treatment (Figure 5, C, D,and K). Associated with the influx of macrophages, there was a synchronous increase in the renal gene expression of inflammatory cytokines. They included TNF-α, IL-1β, and IL-6. The changes in the gene expression were more or less parallel among various cytokines in different strains of mice following GEN treatment (Figure 5, G–I). A notable increase in the expression was seen in WT mice, and likewise, a boosted expression was observed in MIOX-Tg mice following GEN administration, which was more pronounced in the expression of TNF-α. In the realm of inflammatory response, the critical transcription factor that plays a vital role in such pathological processes includes NF-κB. To delineate the role of NF-κB, the status of phosphorylation of its inhibitor (i.e., IκBα) and the transcriptional initiator of its subunit (i.e., p65) were assessed (Figure 5J). The phosphorylation leads to its ubiquitination thereby freeing the NF-κB to enter the nucleus to initiate transcription. In this regard, a marked increase in the expression of phosphorylated IκBα (p-IκBα) was observed in MIOX-Tg mice receiving GEN treatment. A mild increase in the IκBα phosphorylation was observed in WT mice, as well. Likewise, the expression of p-p65 was notably increased in treated MIOX-Tg mice (Figure 5J). On the other hand, a slight decrease in the expression of IκBα and p65 was observed. These observations suggested a remarkable increase in the transcriptional activity of NF-κB in the MIOX-Tg mice.

### MIOX overexpression accentuates GEN-induced renal cellular apoptosis.

**Figure 6:**
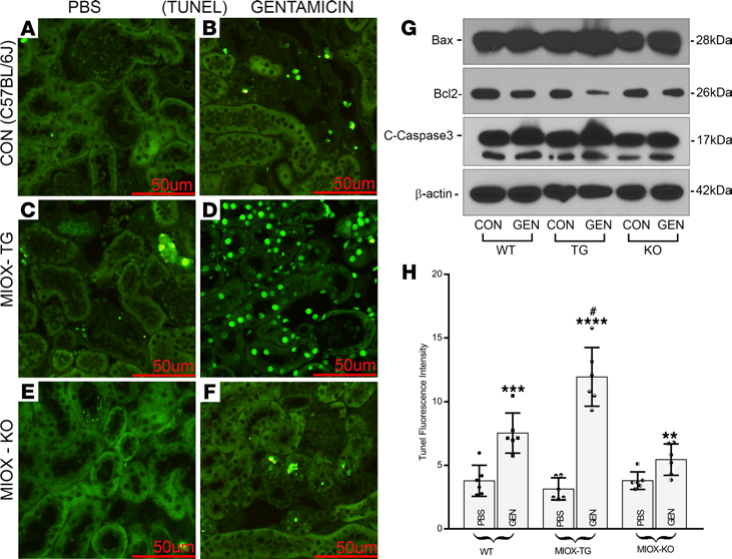
Accentuation of gentamicin-induced renal cellular apoptosis following MIOX overexpression. A mild degree of increased apoptosis was seen in WT mice following gentamicin administration, as gauged by the TUNEL^+^ nuclei (**A**,** B**, and** H**). There was minimal apoptosis in MIOX-KO mice (**E**,** F**, and** H**). A marked apoptosis was noted in MIOX-Tg mice after gentamicin treatment as depicted by quantitation of TUNEL^+^ nuclei (**C**,** D**, and** H**) (*n* = 3 independent experiments with 2 duplicates each; **P ≤ 0.01,*** *P* ≤ 0.001, ****P ≤ 0.0001, compared with control PBS groups; ^#^*P* ≤ 0.01 compared with GEN groups; 1-way ANOVA with Dunn’s multiple-comparison test). Scale bars: 50 μm. By immunoblotting procedures, the changes were seen in the expression of proapoptotic (Bax and cleaved caspase-3) antiapoptotic (Bcl2) molecules following gentamicin administration (**G**). The changes were notably remarkable in kidneys of MIOX-Tg mice receiving gentamicin, indicating that increased MIOX expression accelerates cellular injury.

As mentioned earlier, 4-HNE at low or medium concentrations regulates a wide variety of cell signaling responses and the activity of various transcription factors; however, at high levels, it is likely to induce cell death in the form of apoptosis (24). Moreover, increased or persistent oxidant stress, alluded to earlier, may also contribute to this form of programmed cell death. The extent of apoptosis and the status of molecules (apoptogenic/anti-apoptogenic) that regulate this process was assessed in various strains of mice subjected to GEN treatment. A mild to moderate degree of apoptosis was seen in WT mice following GEN administration, as gauged by the TUNEL^+^ nuclei (Figure 6, A, B, and H). A minimal increase in apoptosis was observed in MIOX-KO mice, suggesting that MIOX gene disruption shields the kidneys from GEN-induced cellular damage (Figure 6, E, F, and H). MIOX-Tg mice showed a marked increase in apoptosis, indicating that MIOX overexpression is highly detrimental to normal cellular homeostasis (Figure 6, C and D). In line with the extent of apoptosis, comparable changes were observed in its modulators (Figure 6G). Immunoblotting analyses revealed a mild-to-moderate increase in the expression of proapoptotic Bax and cleaved caspase-3 in WT mice following GEN treatment, whereas a mild decrease in expression of antiapoptotic Bcl2 was noted. Interestingly, there was a marked decrease in the expression of Bcl2 in MIOX-Tg mice that have undergone GEN treatment. Conversely, a remarkable increase in the expression of proapoptotic Bax and cleaved caspase-3 was observed in MIOX-Tg mice, suggesting that MIOX overexpression accentuates GEN-initiated cellular injury (Figure 6G).

### In vitro evidence of direct modulation of ALOX-12 expression and generation of 12-HETE by GEN in HK-2 cells and, thereby, altered expression of inflammatory cytokines.

**Figure 7:**
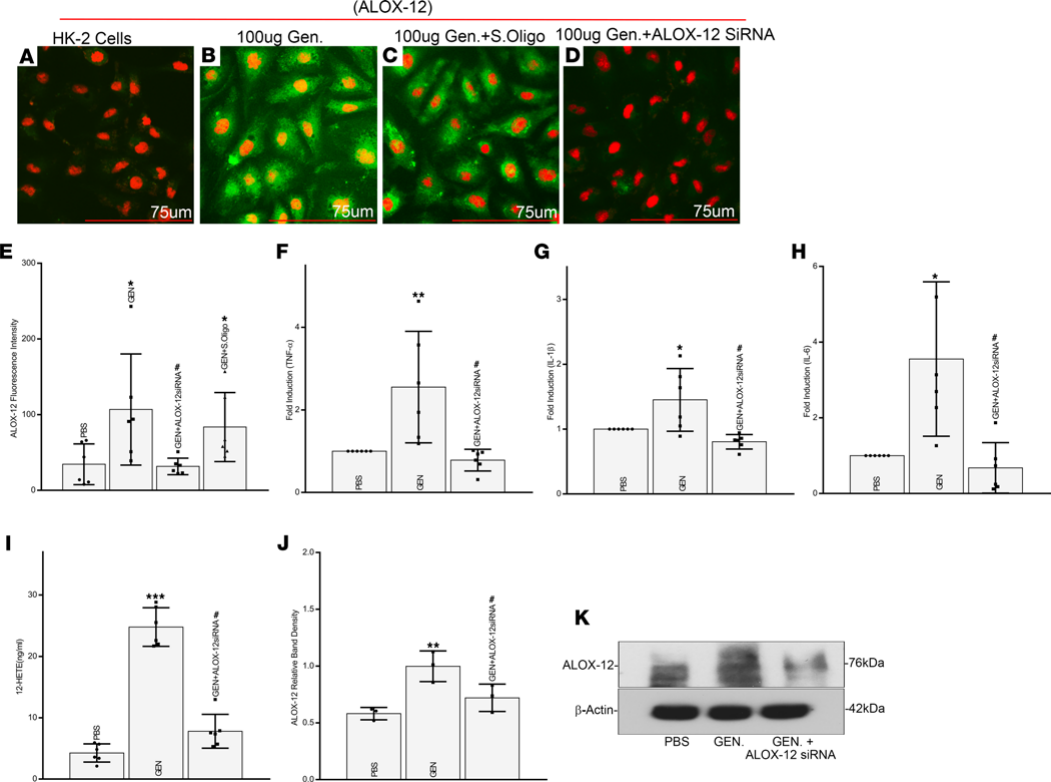
Direct in vitro modulation of ALOX-12 expression and generation of 12-HETE by gentamicin in HK-2 cells with altered expression of inflammatory cytokines. A notable increase in the cytosolic expression of ALOX-12 in HK-2 cells was noted following gentamicin treatment (**A **versus** B**, green). This gentamicin-induced expression was abrogated by ALOX-12 siRNA, and it was unaffected by the scrambled oligo treatment (**C**–**E**). Scale bars: 75 μm. These changes could be replicated by immunoblotting procedures (**J** and** K**). Associated with the ALOX-12 upregulation, there was an increased gene expression of the inflammatory cytokines TNF-α, IL-1β, and IL-6 following gentamicin treatment. Transfection of ALOX-12 siRNA drastically reduced the expression of these cytokines (**F**–**H**) (*n* = 3 independent experiments with 2 duplicates each; *P ≤ 0.05, **P ≤ 0.01, compared with control PBS group; ^#^*P* ≤ 0.01, compared with GEN group; 1-way ANOVA with Dunn’s multiple-comparison test) and generation of 12-HETE in HK-2 cells (**I**) (*n* = 3 independent experiments with 2 duplicates each, ***P ≤ 0.001, compared with control PBS group; ^#^*P* ≤ 0.0001, compared with GEN group; 1-way ANOVA with Dunn’s multiple-comparison test).

To tease out further signaling events, in vitro experiments with HK-2 cells were initiated. First, baseline studies were carried out to confirm the observations made in vivo in various strains of mice. The GEN treatment induced a notable increase in the cytosolic expression of enzyme lipoxygenase (ALOX-12) (Figure 7, A, B, and E) in HK-2 cells. This expression was dampened by ALOX-12 siRNA, while it was unaffected with the treatment of scrambled oligo (Figure 7, C–E). The specific GEN-induced ALOX-12 cytosolic upregulation was also substantiated by immunoblotting studies (Figure 7, J and K). A follow-up question to be addressed was whether or not expression of inflammatory cytokines is associated with the ALOX-12 upregulation. Indeed, the gene expression of all the 3 inflammatory cytokines—i.e, TNF-α, IL-1β, and IL-6—was increased following GEN treatment, and it was specifically related to upregulation of lipoxygenase since transfection of ALOX-12 siRNA notably reduced their expression (Figure 7, F–H). Interestingly, like in various strains of mice, the upregulation of ALOX-12 protein expression was associated with a comparable increase in the generation of 12-HETE following GEN treatment, which was highly attenuated by the concomitant treatment of ALOX-12 siRNA (Figure 7I). These findings suggested the relevance of ALOX-12 as the main executor of GEN-induced cellular perturbations.

### ALOX-12–catalyzed AA metabolite 12-HETE generation promotes GEN-induced bursts of inflammation, as well as activation of the NF-κB transcription factor, with modulation of relevant MAPK cellular signaling kinases in HK-2 cells.

**Figure 8:**
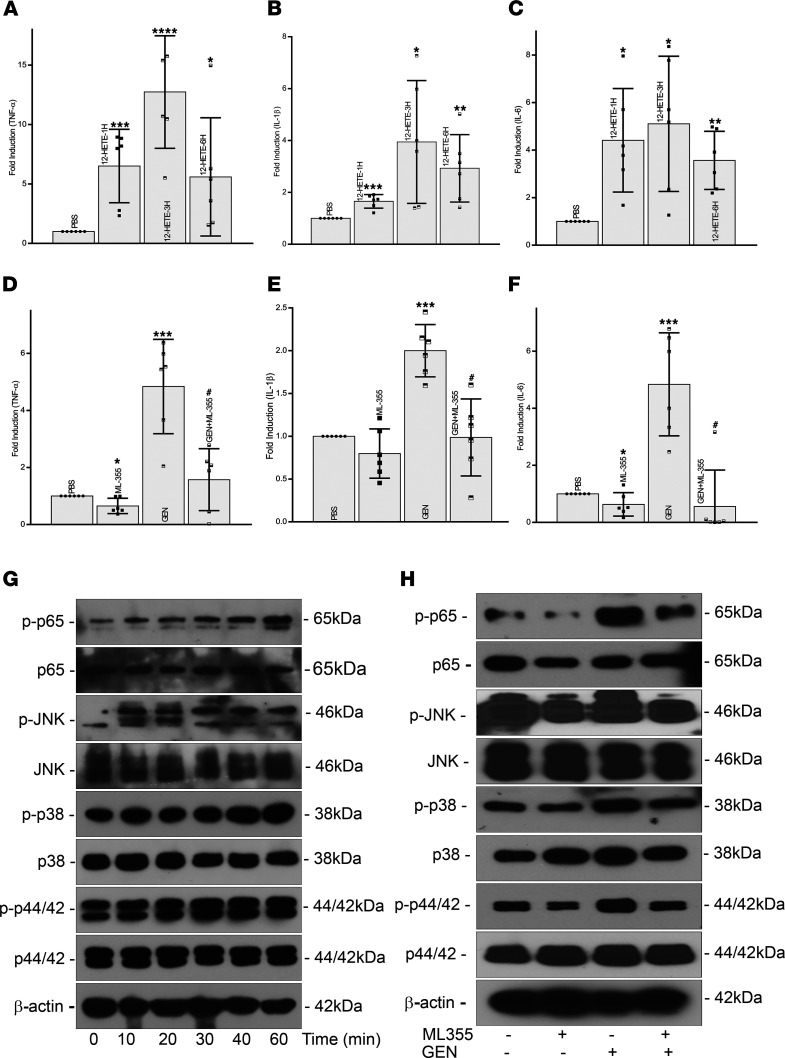
12-HETE promotes gentamicin induced inflammatory cytokines, activation of NF-κB, and modulation of MAPK cellular signaling kinases in HK-2 cells. Treatment of HK-2 cells with 12-HETE caused an increase in mRNA levels of TNF-α, IL-1β, and IL-6; the upregulation could be observed for up to 6 hours (**A**–**C**). The 12-HETE treatment also led to an increase in the phosphorylation of NF-κB subunit p65 and various signaling kinases of MAPK family, including JNK (M_r_ 46 kDa), p38 MAPK, and ERK (M_r_ 44/42 kDa) (**G**). These results indicate that proinflammatory effects of ALOX-12 are mediated via its substrate’s major metabolite, 12-HETE. Along these lines, the HK-2 cells treated with ML355, an inhibitor of ALOX-12, had a notable decrease in the expression of inflammatory cytokines (**D**–**F**, second column). Also, the cotreatment of cells with gentamicin and ML355 notably reduced the gentamicin-induced increased expression of inflammatory cytokines (**D**–**F**, fourth column). Along these lines, a notable decrease was observed in the expression of NF-κB subunit p65 and MAPKs, especially the phosphorylated form of the kinases, with the ML355 treatment (**H**). (*n* = 3 independent experiments with 2 duplicates each; *P ≤ 0.05, **P ≤ 0.01, ***P ≤ 0.001, ****P ≤ 0.0001, compared with control PBS group; ^#^*P* ≤ 0.01, compared with GEN group; 1-way ANOVA with Dunn’s multiple-comparison test).

The HK-2 cells were treated with 100 nM of 12-HETE for 1–6 hours, and relative mRNA levels of genes encoding inflammatory mediators—i.e.*,* TNF-α, IL-1β, and IL-6—were measured. Upregulation was seen at 60 minutes and persisted for up to 6 hours following 12-HETE treatment (Figure 8, A–C). Subsequent immunoblotting experiments were carried out at 60 minutes. During this time frame, the 12-HETE treatment led to a more or less progressive increase in the phosphorylation of NF-κB subunit p65, and inflammatory cytokines or oxidant stress activated various signaling kinases belonging to the mitogen activated protein kinase (MAPK) family, including c-Jun N-terminal kinase (JNK, M_r_ 46 kDa), p38 MAPK (M_r_ 38 kDa), and extracellular signal–regulated kinase (ERK, M_r_ 44/42 kDa) starting at times as early as 10 minutes (Figure 8G). This suggested that the proinflammatory potential of ALOX-12 is exerted via its substrate’s major metabolite, 12-HETE.

To further substantiate the role of 12-HETE in inducing inflammation, HK-2 cells were treated with ALOX-12 specific inhibitor ML355. Being a blocker of ALOX-12, ML355 would attenuate the synthesis of 12-HETE. ML355-treated HK-2 cells revealed a decrease in the expression of inflammatory cytokines—i.e., TNF-α, IL-1β, and IL-6 (Figure 8, D–F; second column). Likewise, cells cotreated with GEN and ML355 significantly reduced the GEN-induced increase in expression of inflammatory cytokines (Figure 8, D–F; fourth column). A drastic decrease was observed in the expression of NF-κB subunit p65 and MAPKs, especially the phosphorylated form of the kinases, with the ML355 treatment (Figure 8H). These results attested to the notion that 12-HETE is the main mediator of inflammatory response signaling in GEN-induced AKI.

### In vivo blocking of 12-HETE production restores cellular integrity, expression of inflammatory cytokines, and renal functional parameters following GEN-induced tubular injury in mice.

**Figure 9:**
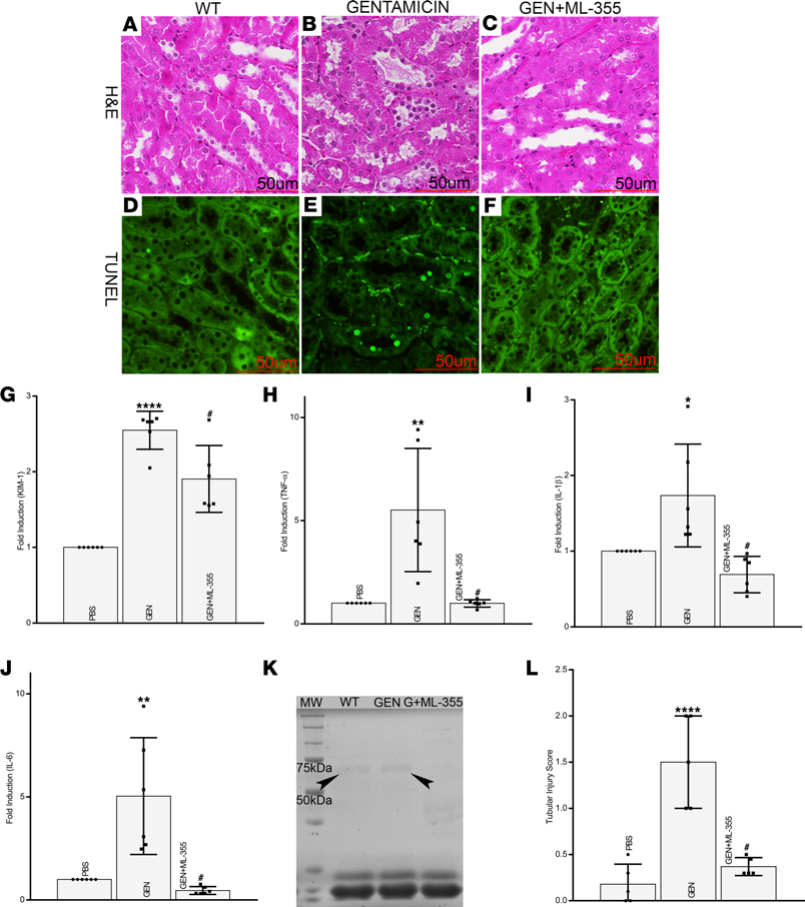
Blocking of 12-HETE restores cellular integrity, expression of inflammatory cytokines, and renal functional parameters following gentamicin-induced injury in mice. The mice receiving gentamicin had cytolysis, vacuolization of epithelia and loss of tubular brush border (**A**,** B**, and** L**). With the concomitant administration of ML355, normal morphological features of tubular epithelia were largely restored (**B**,** C**, and** L**) (*n* = 5; ****P ≤ 0.0001 compared with control PBS group; ^#^*P* ≤ 0.01 as compared with GEN group; 1-way ANOVA with Dunn’s multiple-comparison test). The attenuation of apoptosis can be compared to WT samples (**D**). In addition, the gentamicin-induced cellular apoptosis was reduced with ML355 administration (**E **versus** F**). Scale bars: 50 μm. Also, the expression of marker of tubular renal injury (KIM-1) and inflammatory cytokines was reduced (**G**–**J**) (*n* = 3 independent experiments with 2 duplicates each; *P ≤ 0.05, **P ≤ 0.01, ****P ≤ 0.0001, compared with control PBS group; ^#^*P* ≤ 0.01, compared with GEN group; 1-way ANOVA with Dunn’s multiple-comparison test). Interestingly, urinary protein excretion was restored to normal and gentamicin-induced albuminuria mitigated (**K**). G + ML355, gentamicin + ML355.

To assess if the ALOX-12 inhibitor ML355 has any therapeutic potential, in vivo studies in mice were initiated. ML355 was administered to mice 30 minutes before GEN injection. Mice receiving GEN had cytolysis and vacuolization of epithelia, with loss of tubular brush border, as compared with control mice (Figure 9, A, B, and L). With concomitant administration of ML355, the normal morphology of tubular epithelia was largely restored and had features similar to the control (Figure 9, A, C, and L). Likewise, GEN-induced cellular apoptosis was remarkably reduced with the administration of ML355 (Figure 9, D–F). Along similar lines, expression of kidney injury marker (KIM-1) and some of the inflammatory cytokines were upregulated following GEN treatment, and they were reduced with concomitant treatment of ML355. (Figure 9, G–J). Interestingly, some of the other renal functional parameters, such as urinary protein excretion, were also restored to normal with attenuation of albuminuria following ML355 treatment (Figure 9K).

### 12-HETE mediates tubular injury via GPCR signaling with activation of PKC and phosphorylation of MAPKs and its blocking restores MAPK signaling.

**Figure 10:**
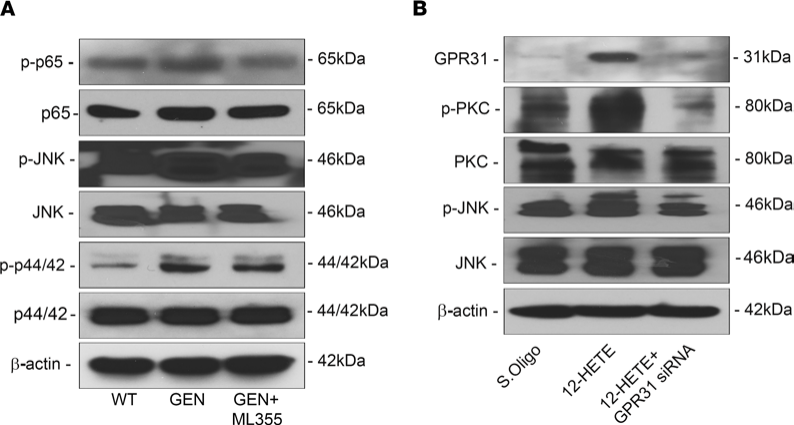
Blocking 12-HETE attenuate MAPK signaling and it uses GPCRs for downstream signaling. The expression of MAPKs was ameliorated following the ML355 treatment, and gentamicin-induced phosphorylation was reduced (**A**). Normally, there is a notable increase in the cellular expression GPR31, along with phosphorylation of PKC and MAPKs—i.e., p-JNK—following 12-HETE treatment in HK-2 cells (**B**). The changes in GPR31, PKC, and JNK were abated in transiently transfected cells with GPR31 siRNA. The above observations suggest a potentially new mechanism related to gentamicin-induced acute tubular injury via the ROS/ALOX-12/12-HETE/GPR31 signaling pathway.

The expression of MAPKs, the downstream signaling molecules, was also attenuated following ML355 treatment. Moreover, their GEN-induced phosphorylation was notably reduced (Figure 10A). To assess if the activation of inflammatory cytokines and sequential events leading to subsequent tubular injury are mediated by GPCRs, in vitro experiments using HK-2 cells were carried out. GPCR signaling has long been implicated in the regulation of signaling pathways related to inflammation, and they serve as receptor for various lipid metabolites (26, 29). Among the well-established GPCRs, we assessed expression of GPR31 since it has high affinity interactions with 12-HETE to elicit a signaling cascade in various cells (26, 30–32). Interestingly, along these lines, we observed a significant increase in the expression GPR31, along with the phosphorylated forms of PKC and MAPKs, following 12-HETE treatment in HK-2 cells (Figure 10B), similar to the increase described in melanoma cells (33). To further, establish that GPR31 is upstream of the PKC and MAPK signaling cascade, HK-2 cells were transiently transfected with GPR31 siRNA. GPR31 gene disruption led to an abolition of the activation of p-PKC/p-JNK signaling (Figure 10B). These results provide insights into one of the seminal mechanisms related to GEN-induced acute tubular injury via *the* ROS/ALOX-12/12-HETE/GPR31 signaling axis.

## Discussion

The results of this investigation indicate that MIOX, renal proximal tubular enzymes, aid in the accentuation of events that trigger oxidant stress; lipid peroxidation; generation of 12-HETE; upregulation of lipoxygenase (ALOX-12); and activation of transcription factors, cytokines, and signaling molecules related to inflammation. Associated with these events, there is also apoptosis and activation of G-protein signaling (GPR31) following 12-HETE treatment. Overall, these sets of events collectively led to progression of GEN-induced AKI confined to the tubules, and this investigation unraveled a potentially novel perturbed cellular signaling pathway involving ROS/ALOX-12/12-HETE/GPR31, which is accentuated by MIOX.

As previously reported in the literature, we also observed kidney injury, mainly confined to the renal proximal tubules (Figure 1) (9, 14, 34). GEN enters the proximal tubules via adsorptive pinocytosis in tubular lumen, as suggested by microdissection techniques (35). However, unlike the report by Huang et al. (8), which suggests tubular injury involving the collecting ducts, we did not observe any significant injury confined to the medullary region in the WT control C57BL/6J mice treated with GEN. Interestingly, the injury was relatively severe in MIOX-Tg mice, and it was confined to the proximal tubules. Conceivably, this segment of the nephron was affected adversely since MIOX is expressed selectively in the proximal tubules; thus, it would be more amenable to GEN-induced injury due to the excessive generation of ROS during MIOX catabolism (23). The accentuated GEN-induced tubular injury was also reflected by the low-molecular-weight proteinuria in MIOX-Tg mice, which may be related to the diminished absorption like other low molecular weight proteins—e.g., cystatin C, NGAL, or KIM-1. The increased albuminuria seen in MIOX-Tg mice may be due the compromise in the receptor-mediated endocytosis of albumin in the excessively injured proximal tubules. This endocytosis, in aggregate, was also reflected in renal functional parameters—i.e., relatively high levels of serum creatinine (Figures 1 and 2).

To assess whether the cellular effects observed are directly related to GEN administration or to its metabolites, in vitro experiments were carried out. Indeed, the GEN treatment of HK-2 cells, a proximal tubular cell line, led to an increased generation of ROS, as assessed by DCF staining (Figure 3). Such an increased generation of ROS has also been reported in other mammalian species (14–16). Interestingly, the generation of ROS was notably boosted in cells transfected with MIOX-pcDNA and concomitantly treated with GEN. Similarly, accentuated generation of ROS has been described in LLC-PK1 kidney cells treated with cisplatin and transfected with MIOX-pcDNA (21). Given the fact that there is an exacerbation of cellular injury in MIOX-Tg mice following cisplatin administration, as well, this would suggest that increased oxidant stress might be a common denominator responsible for AKI affecting the proximal tubules in both the models. In other words, among many others, at least ROS-induced injury may be one of the shared mechanisms by which kidney damage ensues following the administration of GEN or cisplatin.

To delineate other yet-to-be-explored mechanism related to GEN-induced AKI, studies were initiated to assess the extent of lipid hydroperoxidation as a readout of ROS-mediated injury. ROS can adversely affect the biology of plasma membrane lipids, especially the ones with carbon-carbon bonds like PUFA, which happen to serve as a target of oxygen radicals. The end products of such a process include generation of reactive aldehydes, and one of these end products is 4-HNE, which is considered as the major biomarker of lipid hydroperoxidation (24, 25). In line with this dogma, our findings indicate an increased expression of 4-HNE following GEN administration, suggesting that this effect is likely due to the generation of ROS (Figure 4). The idea that this effect is mediated via ROS is reinforced by the observation that there is a significantly higher level of 4-HNE in MIOX-Tg mice with increased expression of MIOX. Interestingly, the ROS modulate MIOX’s transcription with further increased MIOX expression and generation of ROS. The fact that there was a relatively minimal expression in MIOX-KO mice lends further support to this contention. Here, another interesting finding includes the increased granularity of the tubular cytoplasm in MIOX-Tg mice, which may reflect accentuated organelle damage by the ROS-mediated lipid peroxidation (Figure 4, insets). Another pertinent observation of our study includes the upregulation of lipoxygenase (ALOX-12) following GEN treatment, and much more so in MIOX-Tg or transfection of MIOX-pcDNA (Figure 4). It is likely that this increased expression of ALOX-12 is related to the effect of ROS. This contention is well supported by previous studies on eye lens and cornea, which provide direct evidence that oxidant stress induces the upregulation of ALOX-12, conceivably via transcriptional mechanisms (36–38). A follow-up question arises if the increased ALOX-12 expression by acting upon its substrate AA leads to the increased generation of its derivative, 12-HETE. The answer seems to be affirmative. Interestingly, the increased expression of ALOX-12 paralleled the generation of 12-HETE, which apparently was accentuated with the overexpression of MIOX in MIOX-Tg mice. One may question how this spatial localization disconnect leads to renal injury. This may be due to the paracrine effect, as with MIOX overexpression, deposition of fibronectin is increased in the entire tubulo-interstitial compartment (23).

Previous reports in the literature indicate that increased ALOX-12 expression and generation of 12-HETE modulates various pathobiological processes related to inflammation (38, 39). Most of these processes are described in extrarenal tissues, except 1 or 2 reports, which indicate that inhibition of 12/15-lipoxygenase reduces subclinical inflammation in kidneys of diabetic mice (40). With respect to inflammation, these authors also reported reduction in urinary excretion of monocyte chemoattractant protein-1 (MCP-1), which apparently recruits monocytes/macrophages. Interestingly, the landmark publication by Zager and his colleagues reported an increased urinary excretion of MCP-1 in various models of tubular injury, other than GEN, and designated it as one of the biomarkers for the AKI (41). In support of the above observations, we were able to directly visualize the increased monocyte/macrophage influx in kidney tissues of GEN-treated mice, especially the MIOX-Tg (Figure 5). Since 12-HETE modulates inflammatory responses, we assessed the status of various inflammatory cytokines, some of which are secreted by monocytes/macrophage, and they included TNF-α, IL-1β, and IL-6 (42). All these cytokines were upregulated to a variable degree following GEN treatment, especially in the MIOX-Tg mice. One of the important transcription factors related to inflammation and cell survival/death—which it is also activated by 4-HNE—includes NF-κB (24). Its expression was also modulated by the GEN treatment, as reflected by the degree of phosphorylation of its inhibitor, IκBα, and transcription initiator subunit, p65 (43) (Figure 5). The changes in the expression of p-IκBα and p-p65 were overtly seen in the MIOX-Tg mice, suggesting that MIOX is also playing a concordant role in the exacerbation of GEN-induced AKI.

In AKI, one may observe cellular injury associated with different forms of cell death—e.g., ferroptosis, necroptosis, pyroptosis, and apoptosis (4–8, 21). Among them, classical apoptosis, a prototype of programmed cell death, has been well studied for decades, especially in models of ischemia- and cisplatin-induced nephropathy, (5–8, 44, 45). The apoptosis can also be induced by GEN due to the excessively generated oxygen radicals, as alluded to in earlier publications (14–16). Besides excessive ROS, excessively generated 4-HNE, as indicated above, can also can contribute toward the causation of apoptosis, if the cells in vitro are exposed to its higher concentration (24). The present investigation demonstrates that these effects can be seen in in vivo states, as well, where there is an increased generation of 4-HNE (Figure 4) that is associated with an increased degree of apoptosis in the cortical tubules following GEN treatment (Figure 6). Interestingly, our observations also indicate that higher expression of 4-HNE is associated with a relatively severe degree of apoptosis following concomitant treatment of GEN and overexpression of MIOX, for example, in kidneys of MIOX-Tg mice (Figure 6). Such a degree of severity of apoptosis was also observed in AKI secondary to cisplatin exposure (21), thus suggesting that the excessive ROS in MIOX-Tg is most likely responsible for a high degree of cellular damage. Nevertheless, in the present model, the contribution of 4-HNE should be equally recognized. The morphologic evidence of heightened apoptosis in MIOX-Tg mice is also well supported by the changes in the expression of molecules that execute such a process, and these include Bax, Bcl2, and cleaved caspase-3 (Figure 6). Interestingly, similar changes have been reported in these molecules in cisplatin-induced tubular injury in MIOX-Tg mice (21).

Since the involvement of the above-described lipoxygenase pathway in aminoglycoside-induced AKI and modulated by MIOX has been unknown until now, we proceeded to investigate whether ALOX-12 upregulation, generation of 12-HETE, and induction of inflammatory cytokines are specifically modulated by GEN. The data of the in vitro experiments suggest this to be case. Exposure of HK-2 cells to GEN led to increased cytosolic expression of ALOX-12, along with its upregulated protein expression, and these expressions were notably reduced by ALOX-12 siRNA (Figure 7). Along these lines, there was an increased generation of 12-HETE and induction of inflammatory cytokines, and these changes were remarkably nullified by the treatment of ALOX-12 siRNA, suggesting that the activation of these events or pathway are specially related to the GEN-driven effect. Interestingly, the activation of the ALOX-12 pathway has been described in ischemia-reperfusion injury, as well as in other organ systems, such as the liver (26, 31).

The above in vitro studies indicate that ALOX-12 regulates the GEN-induced expression of various inflammatory cytokines, so a follow-up question needs to be addressed is if the metabolite of ALOX-12’s substrate, 12-HETE, can “directly” modulate their expression, as well as the signaling kinases and transcription factors pertinent to inflammation, although the latter is subclinical. The upregulation of ALOX-12 and increased generation of metabolite of AA, 12-HETE, have been implicated in a wide variety of pathobiological processes, including diabetes, where subclinical inflammation is seen in various tissues (38). In support of this contention, we noted that there was an increased expression of various cytokines—TNF-α, IL-1β, and IL-6—following the exposure of HK-2 cells to 12-HETE; it could be seen at 1 hour and persisting up to 6 hours (Figure 8). The upregulation of these cytokines may, in part, be due to the increased expression of NF-κB, a transcription factor that acts as a central mediator during inflammatory processes (27, 28). Moreover, the experiments with ML355, the inhibitor of ALOX-12, would suggest that the 12-HETE modulation is probably upstream of NF-κB–related events (Figure 8). Also, the activation of NF-κB by 4-HNE may be another additive factor in the induction of the upsurge of these cytokines (Figures 4 and 5) (24). Another interesting effect of ALOX-12 and 12-HETE that has been reported in the literature pertains to the increased activity of NADPH oxidase and excessive generation of ROS (38, 46). Under biological stress conditions, the ROS can conceivably induce the activation of various signaling kinases, such as JNK, P38, and P44/42 (47, 48). The findings documented in the current investigation support such biological precepts, where activation of signaling cascade and phosphorylation of kinases in 12-HETE–induced oxidant stress were observed (Figure 8). Furthermore, the fact that the inhibition of ALOX-12 with ML355 reduced the activation of the kinase signaling cascade and phosphorylation of NF-κB, a master regulator of inflammatory cytokines, strengthens the notion that 12-HETE, via ROS, is playing a pivotal role in the initiation of these in vitro events (Figure 8). Here, when discussing the role of ROS, the contribution of GEN per se and MIOX overexpression–induced oxidant stress (Figure 4) should be also taken into account in the worsening of tubular injury.

The next apparent question that needs to be addressed would be if the ALOX-12 inhibitor ML355 has any relevant therapeutic value in the reduction of the GEN-induced tubular injury in an in vivo state. In this regard, there are very few in vivo studies reported in the literature where the effect of ML355 in pathophysiological states has been reported. Zhang et al. demonstrated that ML355 inhibits tissue damage in rhesus macaques in the hepatic ischemia-reperfusion model (26), while Adili et al. described the impairment in vascular thrombus formation with the administration of ML355 (49). Along these lines, the ALOX-12–KO mice showed a decreased expression of inflammatory molecules, including cytokines, chemokines, and the p65 NF-κB subunit in the hepatic ischemia-reperfusion model (27, 31). On the other hand, the ALOX-12–overexpressing primary hepatocytes revealed an increased phosphorylation of the p65 subunit of NF-κB, and a cascade of signaling kinases (i.e., ERK, JNK, and p38 kinases), thus suggesting that lipoxygenase inhibition has some merits in the amelioration of various pathophysiological states in mammals. In view of the fact that we established the relevance of lipoxygenase in the pathobiology of AKI, we proceeded to investigate if the GEN-induced toxic injury and relevant signaling cascade can be suppressed with the administration of ML355. Like other systems, we also observed suppression of events that are caused by GEN-induced toxic injury (Figure 9). Besides the decrease in phosphorylation cascade related to NF-κB and various kinases, as assessed by immunoblotting, we noted a significant reduction in the apoptosis of tubular cells and KIM-1 and the expression of various inflammatory cytokines. Interestingly, the beneficial effects of ML355 were reflected in the renal functional parameters—i.e., amelioration of urinary excretion of albumin (Figure 9 and Figure 10A)—thus establishing a potential therapeutic value of ML355.

In the context of ALOX-12 biology, a downstream effector molecule of 12-HETE—GPR31, a G protein-coupled receptor—has attracted the attention of various investigators (26, 30, 31). GPR31 serves as a high-affinity receptor for 12-HETE; the consensus is that, in the model of hepatic ischemia-reperfusion injury, the effects of 12-HETE are mediated via GPR31. In line with this contention, our experiments with HK-2 cells indicate an upregulation of GPR31 with increased phosphorylation of PKC and JNK kinases by 12-HETE exposure, which was reduced with the GPR31 siRNA treatment (Figure 10B), suggesting that the GEN-induced injury may be mediated via the most downstream effector GPR31 and that these events are also operational in the kidney.

Based on our inference and analyzing results of studies by Zager et al. (50), we would like to mention an interesting analogy. The MIOX-KO mice may rescue the kidney from oxidant injury in ways similar to the iron sucrose (FeS), and protoporphyrin ameliorate prooxidant induced acute renal failure. In this scenario, the authors described the increased expression of a number of cytoprotective genes, and among them, the HO-1 mRNA was the most upregulated. In addition, Sharma et al. (51) indicated that iron chelators attenuate AKI via inhibiting lipid peroxidation and ferroptosis. Our recent study reported similar results in the context of ferroptosis (22). Thus, it would be interesting to investigate the status of cytoprotective proteins in MIOX-KO mice and if they modulate downstream signaling alluded to in this study.

Another renoprotective molecule worth discussing here would be nicotinamide adenine dinucleotide (NAD). In a recent review by Ralto et al., the authors indicate that oxidant stress–mediated renal injury may be due to NAD deficiency (52). It is noteworthy to mention here that perturbations in NAD and NADH have been described in the MIOX catabolic glucuronate-xylulose pathway, and these get accentuated with MIOX overexpression (23, 53). Randomized clinical trials have shown that supplementation of this inexpensive tool (NAD) exerts considerable renoprotective effect. Also, pharmacological interventions, which stimulate nicotinamide phosphoribosyltransferase (NAMPT) activity, or inhibiting NAD-consuming enzymes leads to attenuation of renal injury. Like the amelioration of oxidant stress in the kidney, this simple intervention could also alleviate the cardiac and hepatic ischemia-reperfusion injury.

In summary, one can conclude from the results of in vitro and in vivo experiments of this investigation that the GEN-initiated events (i.e., ROS/4-HNE/ALOX-12/12-HETE/GPR31) modulate the cellular injury in the AKI state, and it is further exacerbated by the overexpression of a renal proximal tubule specific enzyme, MIOX. Also, an integral part of this cascade of events includes the modulation of various inflammatory cytokines and kinases that are as its byproducts, which apparently may also affect the outcome of AKI. Finally, it should be noted that MIOX, lipoxygenase-12, and GPR31 may serve as therapeutic targets to develop small molecules that could be of potential use for betterment of human health, especially in view of the fact that this study utilized HK-2 cells, a well-established human proximal tubular cell line.

## Methods

### Antibodies and reagents.

HK-2 cells, a human proximal tubular cell line, were purchased from the American Type Culture Collection (ATCC). Other reagents were purchased from the following vendors. From Abcam, we used anti–4-HNE (ab46545), and –12(S)-HETE (ab33034) ELISA kit. From Cell Signaling Technology, we used anti-Bcl2 (2876S), –cleaved caspase-3 (9661S), –p-JNK (4668S), -JNK (9252T), –p-NF-κB p65 (3033S), –NF-κB p65 (4764S), –p-IκBα (9246S), –IκBα (4814T), –p-p38 MAPK (4511S), -p38 MAPK (9212S), –p44/42 MAPK (4695S), –p-p44/42 MAPK (9101S), and –p-(Ser) PKC substrate (2261S). From Santa Cruz Biotechnology, we used anti-Bax antibody (sc-526), ALOX-12 siRNA (sc-45984), and GPR31 siRNA (sc-95170). From Origene Technologies, we used siRNA universal control (SICOO1) and MIOX siRNA (SR310776). From Invitrogen, we used TO-PRO-3-iodide (T3605) and Fast SYBR Green Mix (4367659). From MilliporeSigma, we used DMEM, 2’-7’-dichlorofluroescein diacetate (DCF-DA, D6883), GEN sulfate salt (G1264), N-acetyl-L-cysteine (NAC; A7250), and anti–β-actin (A5441), –ALOX-12 (12-lipoxygenase, SAB2100109), and –GPR31 (SAB4501269). From Thermo Fisher Scientific, we used Lipofectamine 2000 Transfection Reagent (11668027); Bio-assay System: Creatinine Assay Kit (DICT-500), Cayman Chemicals: 12(S)-HETE (34570) and ML355 (inhibitor of ALOX-12, 18537). From Bio-Rad, we used anti-F4/80 (MCA497GA). The secondary antibodies were purchased from MilliporeSigma as follows: anti–rabbit IgG horseradish peroxidase (HRP, A0545), –mouse IgG HRP (A9917), –rabbit IgG FITC (F9887), –mouse IgG FITC (F0257), and –rat IgG FITC(F1763). Anti-MIOX antibody was used as previously described (54).

### Cell culture experiments.

HK-2 cells were grown in DMEM containing, 5 mM D-glucose, 10% FBS (Thermo Fisher Scientific), and penicillin-streptomycin solution (100 U/mL penicillin and 100 μg/mL streptomycin; Sigma-Aldrich). They were plated on collagen-coated culture dishes and maintained in a humidified environment with 5% CO_2_ at 37°C. Beside normal HK-2 cells, the pcDNA- MIOX–overexpressing cell line was used for various studies (22, 23). The cells (~2 × 10^5^) were seeded onto 55 cm^2^ culture dishes and maintained to achieve ~80% confluency. Following a brief trypsinization, ~0.5 × 10^5^ cells were plated on the 2.2 cm^2^ coverslips and allowed to attach overnight in DMEM media containing 2% FBS. They were then used for IMF, gene expression, and immunoblotting studies. In addition, the cells were pretreated with NAC (1 mM) for 6 hours and subsequently treated with 100 μg/mL GEN for 24 hours. For another set of experiments, cells were pretreated with ML355 (10 μM) for 30 minutes before subjecting them to GEN or 12-HETE (100 nM) treatment for 10 minutes to 6 hours. For gene disruption studies, HK-2 cells were transiently transfected with MIOX- or ALOX-12 or GPR31 siRNA (50 μM) for 24 hours.

### Animals studies.

Eight-weeks-old male mice were used for the study. The following strains of mice were used: WT C57BL/6J, MIOX-KO, and MIOX-Tg. The generation of mice with overexpression of MIOX (MIOX-Tg) and MIOX-KO (Null) have been described previously in our publications (22, 23). For induction of AKI, mice were injected with 100 mg/kg GEN i.p. daily for 7 days. Control mice (WT) received PBS only. ML355, a specific inhibitor of arachidonate ALOX-12, was injected i.p. to mice 30 minutes before the administration of GEN at a dose of 3 mg/kg. The mice were sacrificed on day 8 following the administration of GEN. Before sacrificing, blood and urine samples were collected for estimation of serum creatinine and assessment of proteinuria. In addition, kidneys were harvested for various morphological studies. Serum creatinine was measured using quantiChrome Creatinine assay kit. The urine samples were centrifuged at 5000*g* for 10 minutes at 25°C. The supernatant was subjected to 12% SDS-PAGE, and the gels were stained with Coommassie Brilliant Blue reagent for the detection of the protein bands.

### Morphological studies and tubular damage scoring.

About 2 mm kidney slices were prepared. Following dehydration in graded series of ethanol, the slices were embedded in paraffin. About 3 μm sections were prepared, mounted on glass slides, heat deparaffinized, and treated with xylene. The tissue sections were rehydrated with decreasing concentrations of ethanol. They were then successively stained with hematoxylin (3 minutes) and eosin (30 seconds). Likewise, sections were processed for PAS staining. The sections were coverslip mounted, and the extent of renal tubular injury score was assessed. The protocol was adapted as previously described by Paller et al. and Guo et al. (55, 56). Briefly, the quantitation of tubular injury score was measured among 6 different groups (WT, WT + GEN, MIOX-Tg, MIOX-Tg+GEN, MIOX-KO, MIOX-KO + GEN) of mice (*n* = 5 in each group). Two independent investigators, one of whom was a pathologist, evaluated the kidney sections stained with H&E and PAS. The entire kidney section was surveyed. Four parameters were used, and they included the following: loss of brush border epithelium, cytoplasmic vacuolization, cytolysis, and basement membrane integrity. The severity of damage was graded from 0 (no injury) to 10 (severe damage) of accumulated values all the 4 parameters.

### Morphological assessment of oxidant stress.

Cells transfected either with pcDNA3.1 or MIOX-pcDNA3.1 were seeded at a density of ~0.5 × 10^5^ on cover slips. They were treated with GEN alone or pretreated with NAC, as indicated above. The treated cells were washed 3 times with PBS for 5 minutes each. The cells were then incubated with 10 μM of 2’, 7’–dichlorofluorescein diacetate (DCF-DA) for 15 minutes at 37°C. They were rewashed twice with PBS. The coverslips were inverted and placed on a glass slide with a drop of DAKO mounting media. The sections were evaluated using an UV microscope equipped with epi-illumination and were photographed. The quantitation of DCF fluorescence was carried out using ImageJ software (NIH) as described previously (57).

### IMF microscopy.

Treated HK-2 cells (~0.5 × 10^5^) were fixed with 4% formaldehyde for 15 minutes at 22°C. They were then washed 3 times with ice-cold PBS. They were permeabilized with PBS-T (PBS + 0.25% Triton X-100) for 30 minutes. They were then immersed in 2% BSA solution in PBS-T for 30 minutes at 22°C. They were incubated with primary ALOX-12 antibody, at a dilution of 1:250, in PBS-T containing 2% BSA for 12–15 hours at 4°C, following which, they were washed 3 times with PBS-T for 5 minutes each at 22°C. They were then incubated with FITC-labeled secondary anti-rabbit antibody for 1 hour. After washing with PBS for 5 minutes, the cells were stained with TO-PRO-3 iodide for 15 minutes to visualize the nuclei (red). The coverslips were then inverted and placed on a glass slide with a drop of DAKO mounting media. Similarly, snap-frozen kidney tissue sections were processed for detection of 4-HNE, F4/80, and MIOX at a dilution of 1:250. Briefly, ~5 mm kidney slices were embedded in OCT mounting media. The OCT blocks were snap frozen at –20°C, and 4 μm cryostat kidney sections were prepared. The sections were air dried and fixed in 10% formalin for 10 minutes at 22°C. After washing with PBS, the sections were successively treated with primary and secondary antibodies. The cells and tissues were evaluated for the expression of various proteins using a UV microscope equipped with epi-illumination. The quantitation of IMF was performed using ImageJ software (57).

### Immunoblot analysis of various proteins.

Kidneys were harvested from various strains of mice. The renal cortices were dissected, diced into 1 mm^3^ fragments, and then homogenized in a RIPA buffer (150 mM NaCl, 1% NP-40, 0.5% deoxycholate, 0.1% SDS, and 50 mM Tris [pH 8.0]) containing protease-phosphatase inhibitor cocktail at 4°C for 30 minutes. In addition, the HK-2 cells were subjected to GEN, ML355, or 12-HETE treatment, while others were concomitantly treated with MIOX, ALOX-12, or GPR31 siRNA. They were gently scraped from the culture dishes with a rubber policeman scraping tool, pelleted, and lysed in RIPA buffer. Following vortexing the cells 5–6 times, they were centrifuged at 12,000*g* at 4°C for 10 minutes. The supernatant was collected for estimation of protein concentration by the Bradford method. The protein concentration was adjusted to 100 μg/mL. Equal amounts of protein (20 μg) from various samples was subjected to 10%–12% SDS-PAGE and transferred to PVDF membranes by electroblotting. The membranes were immersed in 5% nonfat dry milk in TBS-T (Tween-20) at 22°C for 1 hour. They were then individually incubated with various antibodies in 2% nonfat dry milk in TBS-T overnight at 4°C. The membrane blots were washed with TBS-T 3 times for 5 minutes each. They were then incubated with secondary anti-rabbit or -mouse antibodies for 60 minutes at 22°C. Autoradiograms were prepared using West Pico Enhanced ChemiLuminence (ECL) Kit (Thermo Fisher Scientific). The quantitation of band intensity in various blots was carried out using ImageJ software as described previously (57).

### 12(S)-HETE estimation.

The 12(S)-HETE was estimated by using Abcam ELISA kit. First, plasma and standard samples were equilibrated at 22°C and diluted by following the manufacturer instructions. Appropriate diluent (100 μL) was added to nonspecific binding (NSB) wells and B_0_ (wells containing standard diluent, conjugate antibody, and substrate; 0 pg/mL standard) wells of the ELISA plate. Assay buffer (50 μL) was added to NSB wells. Then, 100 μL of prepared standard and diluted samples were added to the appropriate wells. Besides NSB and B_0_ wells, 20 μL of 12(S)-HETE alkaline phosphatase conjugate was added to the standard and sample wells. This was followed by the addition of 50 μL of 12(S)-HETE antibody into B_0_, standard, and samples wells. The ELISA plate was covered with plate sealer, incubated at 22°C for 2 hours, and shaken gently on a rotatory platform at 500 rpm. Well contents were discarded, and the wells were washed 3 times by the addition of 400 μL of 1× wash buffer. After carefully aspirating the residual wash buffer, 200 μL of p-Nitrophenyl phosphate (pNpp) substrate solution was added to each well. The plate was then incubated at 37°C for 3 hours. Absorbance in the samples was read at 405 nm after the addition of 50 μL of stop solution, using an ELISA reader. Total 12(S)-HETE was estimated using a standard curve.

### Quantitative PCR.

Gene expression of various inflammatory genes was assessed using real-time PCR. Briefly, ~10 mg of kidney tissue was used for extraction of RNA by TRIzol method. The RNA was quantified using spectrophotometric readings at 260/280 nm. RNA (1 μg) was used for cDNA synthesis. For quantitative PCR (qPCR), the reaction mixture included 100 ng cDNA, 1 μmol/L each of forward and reverse primers, 1× Fast SYBR Green Master Mix, and 2 μL of nuclease-free water in a total volume of 10 μL. The reaction mixture was subjected to qPCR in an Applied Biosystems Step1Plus Real-Time PCR System Thermocycler. Fold induction (2^ΔΔCt^) was used for estimation of relative expression of target genes. The primers used in this study were as follows. For human β-actin, human forward primer (H-FP): 5′-GGTCATCACCATTGGCAATGAG-3′, human reverse primer (H-RP): 5′-TACAGGTCTTTGCGGATG TCC-3′; for IL-6, H-FP: 5′-GATGAGTACAAAAGTCCTGATCCA-3′, H-RP: 5′-CTGGAGCCA CTGGTTCTGT-3′; for IL-1β, H-FP: 5′-GCTGAGGAAGATGCTGGTTC-3′, H-RP: 5′-TCCATA TCCTGTCCCTGGAG-3′; for TNF-α, H-FP: 5′-CAGAGGGCCTGTACCTCATC-3′, H-RP: 5′-GGAAGACCCCTCCCAGATAG-3′; for mouse β-actin, murine FP (M-FP): 5′-AGACCTCTATGCCAACAC AGTG-3′, M-RP: 5′-ACCGATCCACACAGAGTACTTG-3′; for IL-6, M-FP: CCGGAGAGG AGACTTCACAG-3′, M-RP: 5′-CAGAATTGCCATTGCACAAC-3′; for IL-1β, M-FP: 5′-ACCTGTCCTGTGTAATGAAAGACG-3′, M-RP: 5′-TGGGTATTGCTTGGGATCC-3′; for TNF-α, M-FP: 5′-AGAAGAGGCACTCCCCCAAAAG-3′, M-RP: 5′-TTCAGTAGACAGAAGAGCGTGGTG-3′; and for KIM-1, M-FP: 5′-GGAAGTAAAGGGGGTAGTGGG-3′, M-RP: 5′-AAGCAGAAGATGGGCATTGC-3′.

### TUNEL assay.

GEN-induced DNA damage was assessed by TUNEL procedure. In brief, paraffin-embedded tissue sections were deparaffinized, hydrated with decreasing concentrations of ethanol, and then digested with proteinase K (240 units/mL; Promega Madison) for 15 minutes at 37°C in a humidified chamber. After washing the tissue sections with PBS twice, nicked DNA was enzymatically labeled with a fluorescent nucleotide probe (Roche Applied Science). After placing a drop of DAKO mounting media on the tissue sections, they were coverslip mounted. The extent of apoptosis was evaluated using a UV microscope equipped with epi-illumination.

### Statistics.

Statistical analyses were carried out using GraphPad Prism (version 7.01). The significance was determined using 1-way ANOVA with Dunn’s multiple-comparison test. Results were expressed as mean ± SD of 3–5 samples in each variable.

### Study approval.

All animal procedures used in this study were approved by the animal care and use committee of Northwestern University (no. 2021-2043).

## Author contributions

IS conceptualized, designed, investigated, reviewed, and administered the project and wrote a draft of the manuscript. YL and XZ helped in editing and data curation. YSK provided the resources for research and wrote and edited the manuscript.

## Supplementary Material

Supplemental data

## Version 1 03/22/2022

Electronic publication
