# Supplementary material for: Modulation of gentamicin-induced acute kidney injury by myo-inositol oxygenase via the ROS/ALOX-12/12-HETE/GPR31 signaling pathway
Source: JCI Insight. 2022 Mar 22;7(6):e155487. doi: 10.1172/jci.insight.155487 (PMC8986073; doi:10.1172/jci.insight.155487)
Supplement: Supplemental data [file jciinsight-7-155487-s160.pdf]

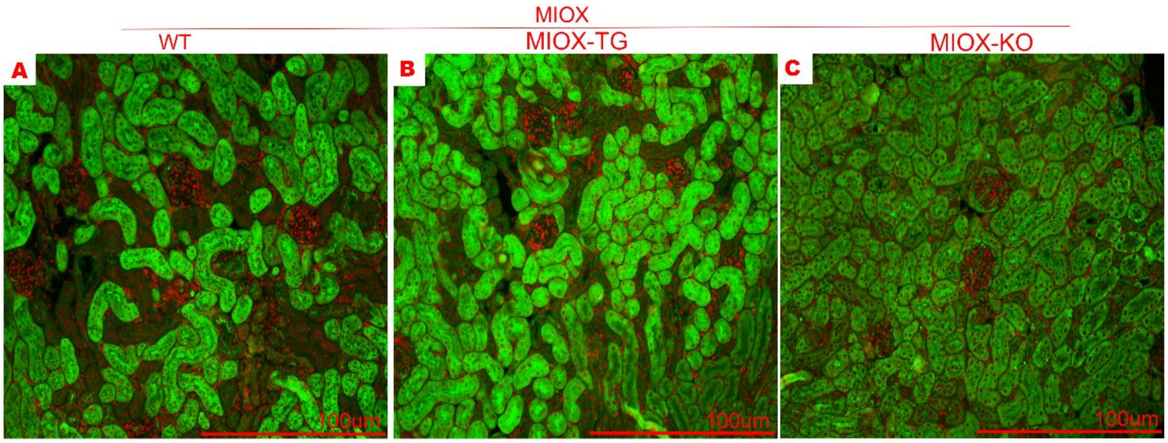

Figure 1: Differential expression of MIOX in various strains of mice. MIOX is expressed in renal cortical proximal tubules (**Panels A vs B**). MIOX-TG mice shows considerable increase in MIOX expression (**Panel B**). A background fluorescence signal is seen in kidney section of MIOX-KO mice (**Panel C**).
